# Dynamic Large-Scale Chromosomal Rearrangements Fuel Rapid Adaptation in Yeast Populations

**DOI:** 10.1371/journal.pgen.1003232

**Published:** 2013-01-24

**Authors:** Shang-Lin Chang, Huei-Yi Lai, Shu-Yun Tung, Jun-Yi Leu

**Affiliations:** 1 Molecular Cell Biology, Taiwan International Graduate Program, Institute of Molecular Biology, Academia Sinica, Taipei, Taiwan; 2 Graduate Institute of Life Sciences, National Defense Medical Center, Taipei, Taiwan; 3 Institute of Molecular Biology, Academia Sinica, Taipei, Taiwan; Washington University School of Medicine, United States of America

## Abstract

Large-scale genome rearrangements have been observed in cells adapting to various selective conditions during laboratory evolution experiments. However, it remains unclear whether these types of mutations can be stably maintained in populations and how they impact the evolutionary trajectories. Here we show that chromosomal rearrangements contribute to extremely high copper tolerance in a set of natural yeast strains isolated from Evolution Canyon (EC), Israel. The chromosomal rearrangements in EC strains result in segmental duplications in chromosomes 7 and 8, which increase the copy number of genes involved in copper regulation, including the crucial transcriptional activator *CUP2* and the metallothionein *CUP1*. The copy number of *CUP2* is correlated with the level of copper tolerance, indicating that increasing dosages of a single transcriptional activator by chromosomal rearrangements has a profound effect on a regulatory pathway. By gene expression analysis and functional assays, we identified three previously unknown downstream targets of *CUP2*: *PHO84*, *SCM4*, and *CIN2*, all of which contributed to copper tolerance in EC strains. Finally, we conducted an evolution experiment to examine how cells maintained these changes in a fluctuating environment. Interestingly, the rearranged chromosomes were reverted back to the wild-type configuration at a high frequency and the recovered chromosome became fixed in less selective conditions. Our results suggest that transposon-mediated chromosomal rearrangements can be highly dynamic and can serve as a reversible mechanism during early stages of adaptive evolution.

## Author Summary

Large-scale chromosomal rearrangements are often associated with dramatic phenotypic changes such as cancer cell formation. It has been speculated that large-scale chromosomal rearrangements may play a crucial role at the early stages of adaptation, since they can quickly change the expression level of multiple genes or even a whole pathway by changing the gene copy number. Nonetheless, it remains unclear whether such mutations can be stably maintained in populations, especially in a fluctuating environment. Here we characterize an adaptive copper-tolerant phenotype in a wild yeast population. We discovered that the adaptive phenotype was contributed to by two large-scale chromosomal rearrangements, which increased the copy number of key components of copper regulation, including a crucial transcriptional activator, Cup2. We further identified three previously unknown downstream targets of Cup2 that also contributed to copper tolerance. Finally, we conducted an evolution experiment to test the stability of the rearranged chromosomes under conditions of relaxed selection. We found that the rearranged chromosomes returned back to the original configuration at a high frequency, and the wild type-like chromosome became fixed in all the evolved cultures. Our results suggest that chromosomal rearrangements can provide a reversible mechanism for cells when adapting to a fluctuating environment.

## Introduction

Organisms have different ways to respond to environmental stresses and evolve corresponding adaptive functions [1]. At the genic level, adaptation can be achieved by subtle, small-scale nucleotide changes (base insertions, deletions or substitutions) that alter gene expression, protein structure or protein interactions. Alternatively, at the genomic level, large-scale genome rearrangements (chromosome duplications, translocations and aneuploidy) create copy number variations that may change gene dosage so as to shape adaptive evolution. Although a similar adaptive phenotype can be achieved by both mechanisms [2], it is still unclear whether one type of mutations is specifically preferred under certain conditions, especially in natural populations.

Unicellular organisms can quickly adapt to different environmental challenges in diverse niches. Comparing different populations of the same microbes that have adapted to distinct environments allows us to identify the underlying mechanisms of adaptive evolution [3], [4]. Studies using the budding yeast *Saccharomyces cerevisiae* have revealed that both small- and large-scale adaptive changes have occurred in natural and laboratory yeast populations [5]. For example, in a natural yeast strain, a few point mutations in the transcriptional factors, *IME1*, *RME1* and *RSF1*, were found to improve sporulation efficiency [6]. Another study in yeast isolated from sherry wines showed that this yeast strain carries two types of mutations in the gene encoding a cell surface glycoprotein. The mutations include a 111-bp deletion in the promoter region that increases its expression level and duplications of a tandem repeat in the coding region that enhance the protein's hydrophobicity [7].

Large-scale changes that involve chromosome duplication, translocation or aneuploidy have been observed in yeast populations during short-term evolution experiments [5], [8]–[13]. Under glucose-limited conditions, evolved strains carry an amplified region that encodes a high-affinity hexose transporter [14]. Under sulfate-limited conditions, amplifications of a high-affinity sulfate transporter locus (*SUL1*) were observed [15]. Beyond changes observed in experimental populations, a chromosome translocation resulting in overexpression of *SSU1*, a gene encoding a sulfite efflux pump, was identified in a sulfate-tolerant wine strain [16]. In other yeast species, such as the clinical isolates of pathogenic *Candida* spp., large-scale chromosomal rearrangements also play an important role in drug resistance. For example, aneuploidy and isochromosome formation increase the copy number and expression of critical genes for fluconazole resistance in *Candida albicans* [17], [18]. Segmental duplications and new chromosome formation were found to be correlated with fluconazole tolerance in *Candida glabrata* [19]. These studies indicate that large-scale changes allow yeast to quickly adapt to different environments. Despite this wealth of experimental data, it is less clear how cells maintain these mutations over a long evolutionary timescale since large-scale rearrangements are often accompanied by extra costs. In sexual populations, large-scale rearrangements can also result in gamete lethality when they are heterozygous unless they localize near the telomeres and do not carry regions with essential genes [20].

The mutations that cause large-scale chromosomal rearrangements occur at a high frequency in yeast populations. In mutation-accumulation lines of haploid budding yeast, the estimated spontaneous mutation rate of large-scale changes was 4.8-fold higher than that of small-scale changes (0.019 and 0.004 per genome per cell division, respectively) [21]. In another similar experiment in diploid yeast cells, it was shown that most structural variations occurred in the subtelomeric regions [22]. Like other types of mutations, most large-scale changes are probably deleterious and will quickly vanish from the population [23]. However, even in large evolving populations isochromosome formation and segmental duplication can be detected after as few as 5 or 100 generations, respectively [24], indicating that large-scale mutations supply the population with genetic variation that could facilitate adaptation to novel environments.

It has been suggested that Ty transposons may play an important role in the formation of large-scale chromosomal changes in yeast [25]. Although the yeast genome is relatively compact compared to other eukaryotic genomes, about 1–4% of the yeast genome is comprised of Ty sequences [26]. In addition, Ty sequences are often found in clusters [25]. Inverted arrays of transposon sequences can cause replication fork stalling that leads to chromosome breakage, especially when the replication machinery or checkpoints are compromised [27]–[29]. Those Ty-rich regions may constitute a preferred double-strand break site similar to the fragile sites observed in mammalian chromosomes [28], [30]. Previous studies in budding yeast suggested that many observed chromosomal rearrangements might result from ectopic recombination between Ty sequences [14], [30]–[33]. It is likely that Ty sequences often serve as initiation sites for generating chromosomal rearrangements.

Our knowledge about natural adaptation of budding yeast is often complicated due to human interference in the natural history of yeast. Yeast strains collected from Evolution Canyon (EC) provide an excellent model for studying how yeast populations adapt to natural environments. EC is an east-west-oriented canyon at Lower Nahal Oren, Israel, that originated 3–5 million years ago and is believed to have experienced minimal human disturbance [34]. Its microclimates provide ideal conditions for diverse local adaptations of many organisms [35]–[37]. In previous work, we employed a panel of phenotypic assays to characterize 14 diploid yeast strains collected from different locations within EC. We observed that a specific group of EC yeast strains (EC-C1) could tolerate a high concentration of cadmium. The cadmium-resistant phenotype was shown to be caused by an ancient allele of *PCA1* (*PCA1-C1*), which encodes a metal efflux pump [38].

Here, we show that the same group of EC yeast strains was also highly resistant to another metal, copper. However, the copper-tolerant phenotype is not correlated with the *PCA1-C1* mutant allele. Instead, the copper-tolerant phenotype mainly results from chromosomal rearrangements that increase the copy numbers of *CUP1* and *CUP2*, two major genes involved in copper regulation [39], [40]. By analyzing the whole-genome expression pattern of cells carrying different copy numbers of *CUP2*, we found three previously unidentified genes, *PHO84*, *SCM4* and *CIN2*, whose expression was regulated by Cup2 dosage and contributed to copper tolerance. Finally, we observed that the chromosomal rearrangements in EC-C1 cells were highly reversible. When cells were growing in medium with 1 mM of copper sulfate, a wild type-like chromosome reappeared and was fixed in the population within 300 generations. These results suggest that large-scale chromosomal rearrangements provide not only a fast arising but also readily reversible source of variation during early stages of adaptive evolution.

## Results

### One subset of EC diploid strains is highly tolerant to copper

**Figure 1:**
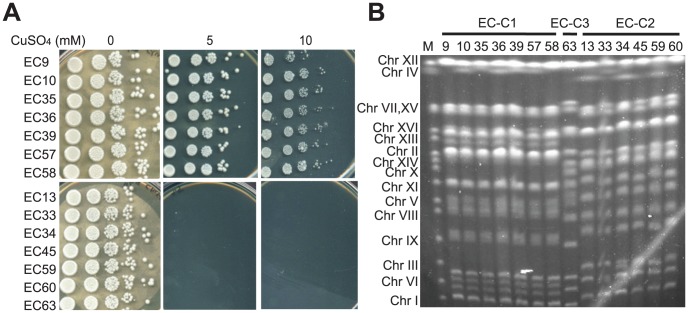
A subset of EC diploid strains is highly tolerant to copper. (A) Strains EC9, 10, 35, 36, 39, 57 and 58 can tolerate a high level of copper (10 mM CuSO_4_). Diploid EC strains were grown in YPD media overnight, serially diluted and plated on YPD plates containing different concentrations of CuSO_4_. Plates were incubated at 28°C until obvious colonies were formed. (B) Pulsed-field gel electrophoresis analysis reveals that the EC strains comprised three major karyotypes: EC cluster 1 (EC-C1) containing seven strains (EC9, 10, 35, 36, 39, 57 and 58), EC cluster 2 (EC-C2) containing six strains (EC13, 33, 34, 45, 59 and 60), and EC cluster 3 (EC-C3) containing only one strain (EC63). All copper-tolerant strains belong to EC-C1. M, yeast chromosomal DNA from a standard laboratory strain.

Yeast strains collected from Evolution Canyon have been shown to adapt to various environmental stresses, such as oxidative stress, UV radiation, and high concentrations of cadmium [37], [38], [41]. In addition, most of the EC strains are heterothallic [42]. To further examine if EC strains have evolved other adaptive phenotypes, we tested the growth of EC diploid strains on several metal-containing plates. Interestingly, those cadmium-resistant strains (EC-C1 strains, including EC9, 10, 35, 36, 39, 40, 57 and 58) could also tolerate high concentrations of copper sulfate (Figure 1A). However, when we crossed the copper-tolerant haploids with a copper-sensitive strain and analyzed the meiotic products, we found that the copper-tolerant phenotype did not co-segregate with the *PCA1-C1* mutation responsible for the cadmium resistance (data not shown). In our previous study, we also showed that the *PCA1-C1* allele did not increase the copper tolerance when it was put into a copper-sensitive strain [38]. Together, these results suggest that other genes are responsible for the tolerance to copper in the EC-C1 strains.

In our previous study, we observed that the diploid *S. cerevisiae* strains isolated from Evolution Canyon comprised three major karyotypes (with some minor deviations), including EC cluster 1 (EC-C1), EC cluster 2 (EC-C2) and EC cluster 3 (EC-C3) (Figure 1B) [38]. This karyotype clustering pattern is consistent with the results from the phylogenetic analyses [42], [43]. Because all copper-tolerant strains belong to EC-C1, it suggests that the metal-tolerant phenotypes had already evolved before the EC-C1 populations split. Therefore, we chose EC9 from EC-C1 as representative of this clade for subsequent genetic analyses.

### The copper-tolerant strains have gross chromosomal rearrangements

Laboratory evolution experiments have shown that chromosomal rearrangements can result in adaptive changes to gene copy number [14], [15], [44]. To further examine each individual chromosome, chromosomes separated by pulsed-field gel electrophoresis (PFGE) were subjected to Southern blotting using chromosome-specific DNA probes. The result showed that EC-C1 strains have high chromosome heterozygosity. They carry at least four heterozygous chromosome pairs (chromosomes 5, 6, 8 and 14), as revealed by length differences between homologous chromosomes. In addition, we observed several large chromosomal rearrangements in EC-C1 strains that had resulted in an elongated chromosome 10, an elongated chromosome 8 of almost twice its original size, and a novel chromosome that was hybridized by probes from both chromosomes 7 and 8 (Figure S1).

**Figure 2:**
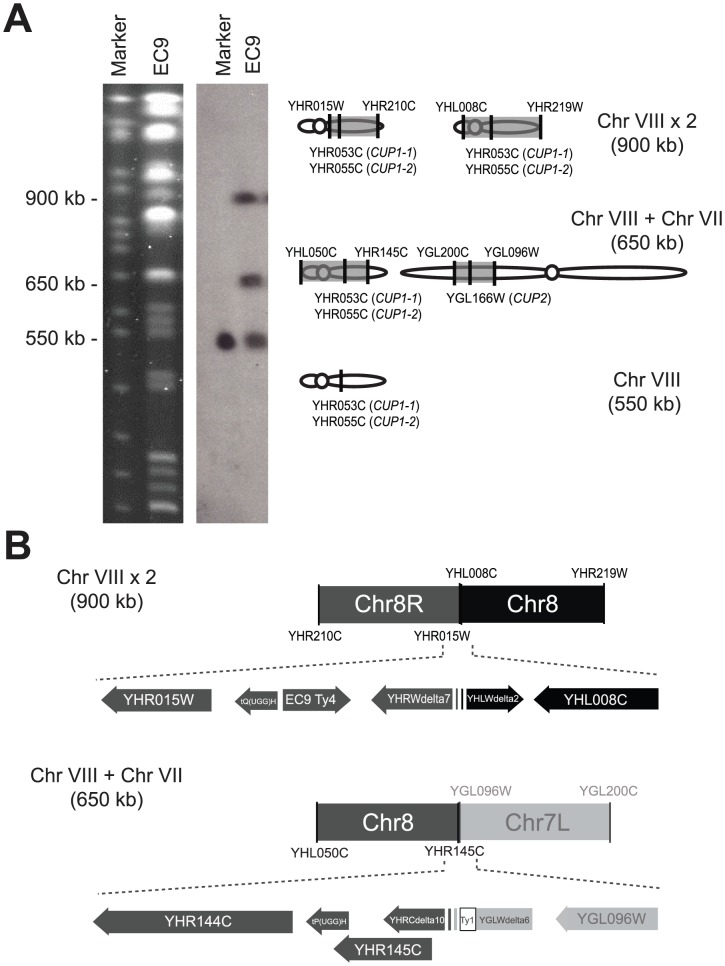
EC-C1 strains contain large-scale chromosomal rearrangements. (A) A diagram showing the structures of the rearranged chromosomes 8 in EC-C1 strains. EC-C1 strains contain three chromosomes that can be hybridized by a chromosome 8 probe (left panel and Figure S1). These chromosomes were isolated and subjected to array CGH analysis. The results showed that the 900-kb chromosome was a fusion product of two chromosome 8 fragments (between *YHR015W* to *YHR216W* and *YHL008C* to *YHR219W*) while the 650-kb chromosome contained a chromosome 7 fragment (between *YGL096W* and *YGL200C*) and a chromosome 8 fragment (between *YHL050C* and *YHR144C*). The 550-kb fragment is a wild type copy of chromosome 8. The shaded areas are the fragments contained in the rearranged chromosomes. The positions of the *CUP1* and *CUP2* genes are also indicated. (B) The junction sites of two rearranged chromosomes. The 900-kb chromosome was formed by fusing the regions near *YHR015W* and *YHL008C*. The 650-kb chromosome was formed by fusing the regions near *YHR145C* and *YGL096W*. EC9-Ty4, a Ty4 sequence found only in EC-C1 strains. Ty1, a Ty1 sequence found only in the junction site. The detailed DNA sequences can be found in Figure S2 and in GenBank under the accession numbers JX101633 and JX101634. The scale of this illustration is not proportional to the base pair size of genes.

**Figure 3:**
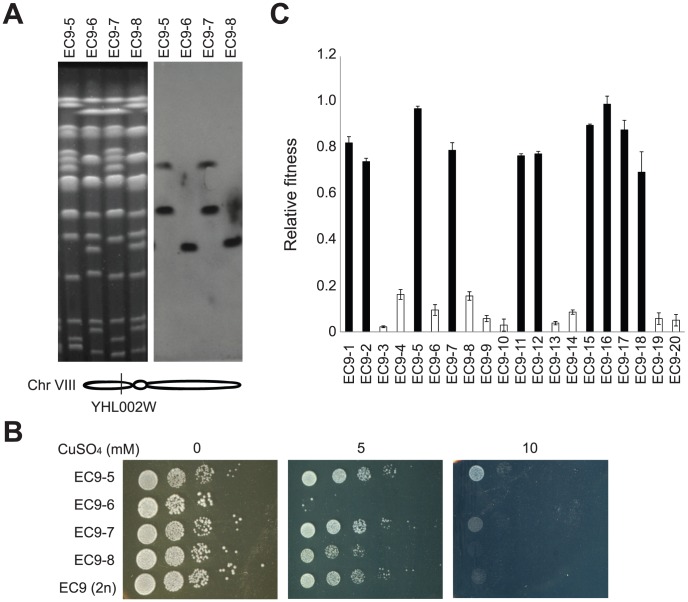
Haploid segregants of EC9 carrying rearranged chromosomes are more tolerant to copper. (A) EC9-5, EC9-6, EC9-7 and EC9-8 are haploid segregants from a single EC9 tetrad. Two of them (EC9-5 and -7) carry two rearranged copies of chromosome 8, and the other two carry a wild type copy of chromosome 8. A gene (*YHL002W*) located on the left arm of chromosome 8 was used as a probe for Southern blotting. M, yeast chromosomal DNA from a standard laboratory strain. (B) Segregants carrying the rearranged chromosomes are more copper tolerant. (C) Rearranged chromosomes are responsible for about 60% of the observed copper-tolerant phenotype. Twenty EC9 haploid segregants were grown in medium with or without 1.5 mM CuSO_4_ and their growth rates were measured. Relative fitness represents the ratio of cell growth rate in medium with 1.5 mM copper to that in medium without 1.5 mM copper.

The fact that the latter two chromosomal rearrangement events that we observed both involved chromosome 8 prompted closer examination. The rearranged chromosomes were purified from PFGE gels and subjected to array-based comparative genomic hybridization (aCGH) using *S. cerevisiae* oligonucleotide microarrays. These experiments revealed that the aberrant 900-kb chromosome 8 is a fusion product of two chromosome 8 fragments (between *YHR015W* to *YHR210C* and *YHL008C* to *YHR219W*) and that the novel 650-kb chromosome is a fusion product of a small chromosome 7 fragment (between *YGL096W* and *YGL200C*), a large chromosome 8 fragment (between *YHL050C* and *YHR145C*) and the telomere of chromosome 8 (between *YHR210C* to *YHR217C*) (Figure 2). We also conducted aCGH using genomic DNA isolated from EC-C1 diploid cells (EC9) and haploid cells that carry both rearranged chromosomes (EC9-7 in Figure 3B). The results confirmed that the copy numbers were indeed increased in the duplicated regions.

### Breakpoints of the rearranged chromosomes contain many transposon sequences

To understand how novel chromosomes were formed, we fine-mapped the junction sites of the rearranged chromosomes. We designed primers near each possible breakpoint according to the aCGH data (i.e., regions close to *YHR015W*, *YHR210C*, *YHL008C*, *YHR219W*, *YHL050C*, *YHR145C*, *YGL200C* and *YGL096W*) and used these primers to find out the junction site of two chromosomal fragments (see Materials and Methods). As shown in Figure 2C and Figure S2, the aberrant 900-kb chromosome 8 was formed by fusing the regions near *YHR015W* and *YHL008C* and the novel 650-kb chromosome was formed by fusing the regions near *YHR145C* and *YGL096W*. Interestingly, we found that the breakpoints were all flanked by Ty sequences (next to *YHR015W*, *YHL008C*, *YHR145C* and *YGL096W*), indicating that transposable elements might be the mediator of these chromosomal rearrangements. Moreover, three out of the four flanking regions (*YGL096W*, *YHR015W* and *YHL008C*) contain multiple Ty long terminal repeats (LTRs) including at least one inverted LTR pair. It is possible that the double-strand break hotspots formed in these Ty arrays allow chromosomes to be rearranged at a high frequency. We also sequenced the breakpoints of the other EC-C1 strains. The result confirmed that the same chromosomal rearrangements exist in all EC-C1 strains (Table S1).

### The copper-tolerant phenotype is correlated with chromosomal rearrangements

To assess the contribution of the rearranged chromosomes to copper tolerance, we dissected tetrads (meiotic products) of EC9 and measured the copper resistance of individual haploid segregants (spores). The spore viability of EC9 is about 60% due to the chromosomal rearrangement of EC-C1 strains. In eight four-viable-spore tetrads yielding 32 haploid segregants, all sixteen segregants containing both rearranged chromosomes showed higher copper tolerance than the other sixteen segregants containing only wild type copies of chromosomes (Figure 3 and Figure S3). However, we noticed that within these two groups of segregants, there were different levels of copper tolerance between individual clones, indicating that the copper-tolerant phenotype in EC-C1 strains was polygenic and genes on other chromosomes might also be involved in copper tolerance with minor effects. We measured the relative fitness of twenty EC9 haploid segregants in copper-containing medium (Figure 3C). By comparing the fitness between stains carrying rearranged chromosomes and the wild type chromosome, we estimated that rearranged chromosomes are responsible for about 60% of the observed copper-tolerant phenotype.

**Figure 4:**
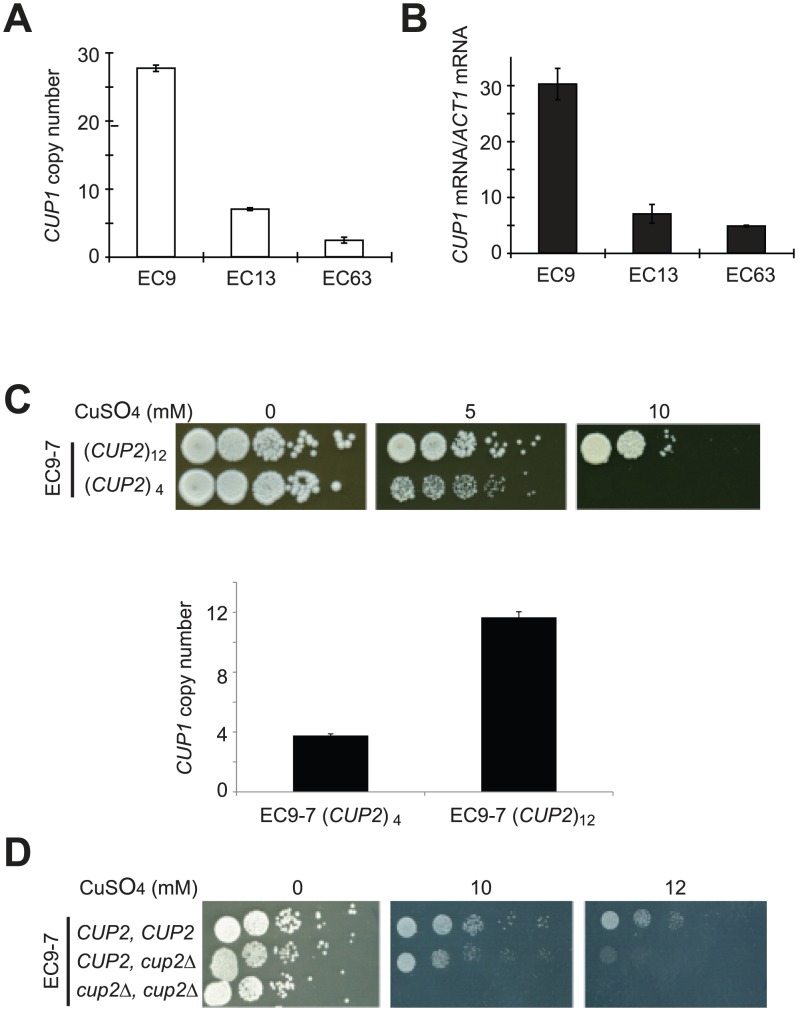
Increased copy numbers of *CUP1* and *CUP2* were correlated with enhanced copper tolerance in EC-C1 strains. (A) The copy number of *CUP1* in EC9 (an EC-C1 strain) is higher than that in EC13 (an EC-C2 strain) and EC63 (an EC-C3 strain). Total genomic DNA was isolated from EC9, EC13 and EC63, and subjected to quantitative PCR using CUP1-specific and ACT1-specific primers. The *CUP1* DNA levels were normalized to the *ACT1* DNA levels. (B) EC9 has a higher *CUP1* expression level after copper treatment. EC9, EC13 and EC63 log-phase cells were treated with 1 mM CuSO_4_ for 2 h. Total RNA was isolated from the Cu-treated cells, reverse-transcribed and subjected to quantitative PCR using CUP1-specific and ACT1-specific primers. The *CUP1* mRNA levels were normalized to the *ACT1* mRNA levels. (C) Amplification of *CUP1* has a major contribution to copper tolerance in EC-C1 cells. Haploid cells carrying different copy numbers of *CUP1* were grown in YPD media overnight, serially diluted and plated on YPD plates containing different concentrations of CuSO_4_. Total genomic DNA was isolated from EC9-7 and EC9-7 with *CUP1* deletion, and subjected to quantitative PCR as previously described. (D) Copper tolerance is correlated with the copy number of *CUP2*. Haploid cells (EC9-7) carrying two, one or no copies of *CUP2* were grown in YPD media overnight, serially diluted and plated on YPD plates containing different concentrations of CuSO_4_.

When the rearranged chromosomes were inspected, we observed that genes involved in response to copper ions (*CUP1*, *CUP2* and *COX23*) were significantly enriched. *CUP1* is a gene encoding a metallothionein and its expression level has been shown to play an important role in copper tolerance [39]. We measured the *CUP1* gene copy number and expression level using quantitative PCR. The results showed that the *CUP1* copy number and mRNA level in EC9 (an EC-C1 strain) were about 5–6-fold higher than expression in EC34 and EC63 (EC-C2 and EC-C3 strains) after cells were treated with CuSO_4_ (Figure 4A and 4B). To confirm that the increased copies of *CUP1* are important for copper tolerance in EC-C1 strains, we deleted eight copies of *CUP1* in an EC9 haploid segregant (EC9-7 in Figure 3B) and measured their copper sensitivity. The results showed that cells with fewer copies of *CUP1* were indeed less copper-tolerant (Figure 4C).

Cup2 is a copper-binding transcriptional factor that activates *CUP1* expression [40], [45]. The chromosome rearrangements increase the *CUP2* copy number to three in EC-C1 cells. To determine whether the increased copy number of *CUP2* contributes to copper tolerance in EC-C1 strains, we used EC9-7 to construct yeast strains carrying zero, one or two copies of *CUP2* and tested their copper sensitivity. The sensitivity of cells to copper was negatively correlated with the copy number of *CUP2* (Figure 4D), indicating that an increase in the copy number of *CUP2* is also important for copper tolerance. Our results indicate that amplification of both *CUP1* and *CUP2* genes are required for cells to achieve high copper tolerance.

### *PHO84*, *SCM4*, and *CIN2* are involved in copper tolerance in EC-C1 strains

The dosage effect of *CUP2* prompted us to investigate the downstream targets of the Cup2 transcription factor. In previous studies, Cup2 has been shown to regulate three metal-responsive genes, including two metallothionein genes, *CUP1* and *CRS5*, and the copper-zinc superoxide dismutase gene *SOD1* [40], [45]–[47]. To identify more candidate genes under the regulation of Cup2, we performed a whole-genome expression pattern analysis of the EC9 haploid segregant carrying different copy numbers of *CUP2* (EC9-7, EC9-7 cup2Δ and EC9-7 cup2Δ cup2Δ in Figure 4D). Cells were treated with 1 mM CuSO_4_ for 1 h, which resulted in no obvious effects on the growth of *cup2* null mutant cells. Total RNA from treated samples was collected and analyzed using microarrays. In *cup2* double deletion cells we found 39 genes with reduced (1.5-fold or more) expression compared with wild type cells (Table S2). Among these genes, 18 showed a positive correlation between their expression levels and the copy number of *CUP2*.

**Figure 5:**
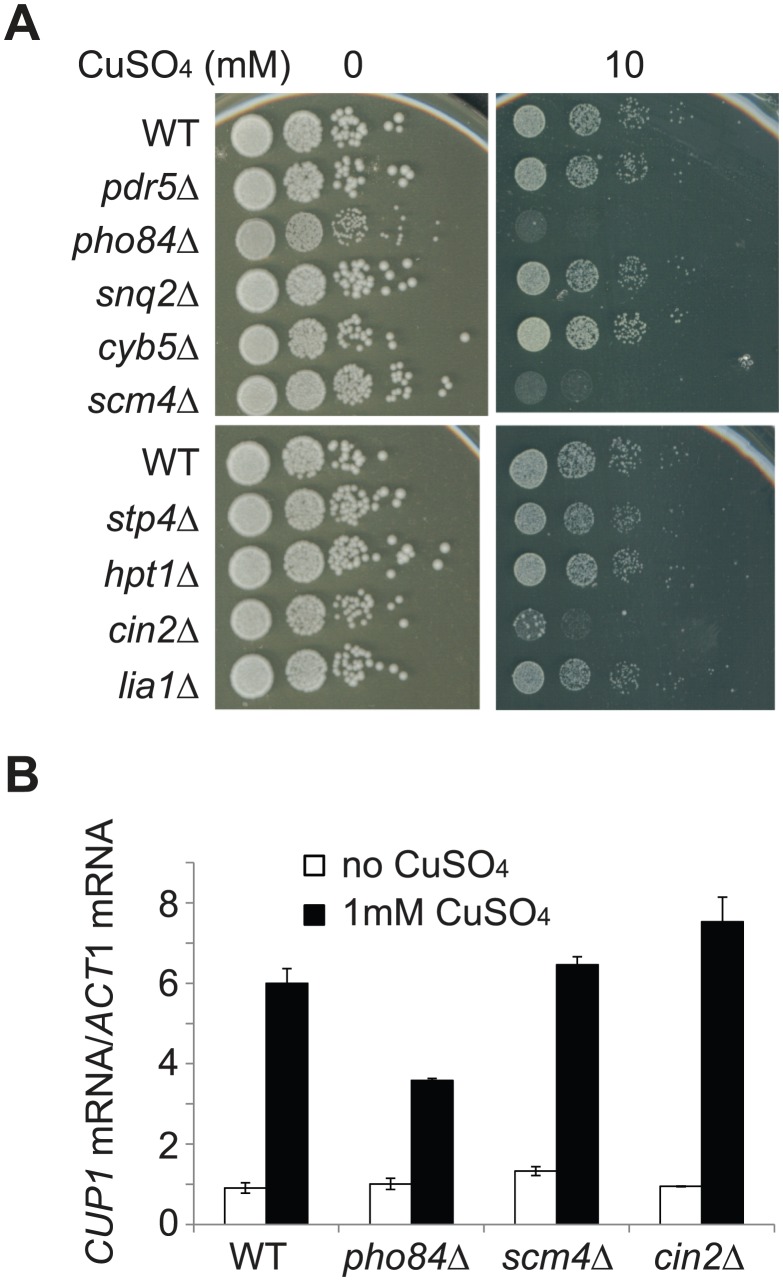
*PHO84*, *SCM4*, and *CIN2* are involved in copper tolerance. (A) Eight non-essential genes (*PDR5*, *SNQ2*, *CYB5*, *SCM4*, *STP4*, *HPT1*, *CIN2*, and *LIA1*), whose expression levels correlated with their *CUP2* copy number, and *PHO84*, whose expression showed more than a 3-fold difference between wild type and *cup2* double deletion cells but did not correlate with *CUP2* copy number, were tested for their contributions to copper tolerance. Individual genes were deleted in the EC9-8 haploid background (WT). Cells were grown in YPD media overnight, serially diluted and plated on YPD plates containing different concentrations of CuSO_4_. (B) *CUP1* expression is reduced in *pho84Δ* cells. Total RNA was isolated from cells with or without copper treatments, reverse-transcribed and subjected to quantitative PCR using CUP1-specific and ACT1-specific primers. The *CUP1* mRNA levels were normalized to the *ACT1* mRNA levels.

To directly test the effect of these candidate genes on copper tolerance, we examined eight non-essential genes in the aforementioned group (*PDR5*, *SNQ2*, *CYB5*, *SCM4*, *STP4*, *HPT1*, *CIN2* and *LIA1*) and *PHO84*, a gene whose expression showed more than a 3-fold difference between wild type and *cup2* double deletion cells but did not correlate with the *CUP2* copy number. We found that when individual genes were deleted, three mutant strains (*pho84Δ*, *scm4Δ* and *cin2Δ*) showed reduced tolerance to copper sulfate (Figure 5A). To rule out the possibility that the mutant cells were sensitive to sulfates instead of copper, the deletion strains were also tested on plates containing copper chloride (Figure S4). Our result indicates that *pho84Δ*, *scm4Δ* and *cin2Δ* mutant cells were indeed sensitive to copper despite no reported linkage between these genes and copper tolerance. We further tested the effect of increased expression of these three genes by introducing a CEN plasmid carrying *PHO84*, *SCM4* or *CIN2* into EC9-8 haploid cells and examining their copper tolerance. However, we were unable to detect obvious growth differences using spot assays (data not shown). We also tried to measure cell fitness using a more sensitive competitive fitness assay, but it was unsuccessful as the EC cells tended to clump together in copper-containing medium. Our results suggest that *PHO84*, *SCM4* and *CIN2* are involved in copper tolerance, but it is less clear how much the increased expression of these three genes contributes to the elevated copper tolerance in EC-C1 cells.

To investigate the molecular mechanisms about how *PHO84*, *SCM4* and *CIN2* affect copper tolerance, we tested whether expression of the *CUP1* gene was affected by mutations in these three genes. Total RNA was isolated from cells treated with 1 mM CuSO_4_ and the expression level of *CUP1* was measured using quantitative PCR. In *pho84Δ* cells, *CUP1* expression was significantly reduced, suggesting that Pho84 may influence copper tolerance through a Cup1-dependent mechanism (Figure 5B).

### Rearranged and wild-type chromosomes share the same sequence

In yeast, it has been shown that large-scale chromosomal rearrangements occur frequently and beneficial ones can become fixed rapidly in the population [14], [21], [24]. It is therefore of interest whether the observed rearranged chromosomes have existed in the EC strains for a long time or have been formed recently. We sequenced a 6628-bp fragment (corresponding to positions 188,179 to 194,679 bp on chromosome 7) from the rearranged chromosome 7 and a 6602-bp fragment (corresponding to positions 496,154 to 502,755 on chromosome 8) from the aberrant chromosome 8 and compared them with wild-type chromosomes (see Materials and Methods for details). Both fragments had the same sequence as that on wild-type chromosomes. From the genome sequence divergence between two closely related yeast species, *S. cerevisiae* and *S. paradoxus*, we obtained an estimation that it may require about 0.25 million years to accumulate 1% of sequence divergence in intergenic regions [48]. If we applied this estimation to the case of the rearranged chromosomes in EC-C1 strains, it suggests that the rearranged chromosomes occurred in the last 3800 years assuming that there was no recombination between the rearranged and wild type chromosomes.

### Rearranged chromosomes revert to wild-type-like chromosomes in laboratory evolution experiments

**Figure 6:**
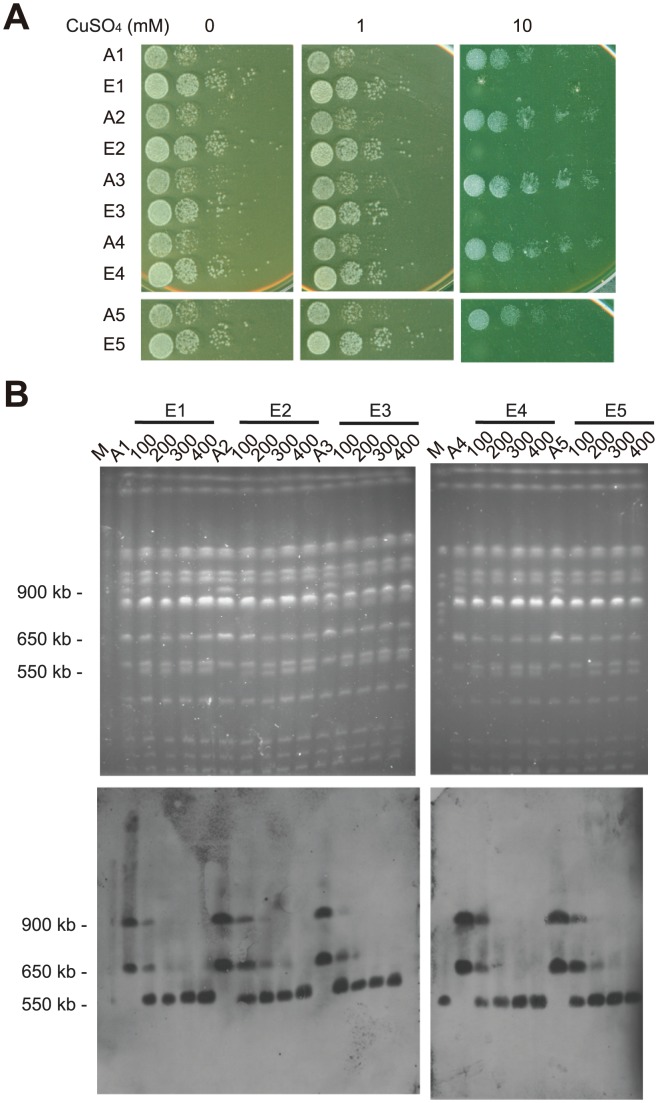
Rearranged chromosomes revert back to wild-type-like chromosomes in laboratory evolution experiments. (A) Experimentally evolved cells exhibited improved growth on YPD or YPD with 1 mM CuSO_4_ but decreased their tolerance to high concentrations of copper after 400 generations of growth in medium containing 1 mM CuSO_4_. Ancestral cells (A1–A5) and evolved cells (E1–E5) were grown in YPD media overnight, serially diluted and plated on YPD plates containing different concentrations of CuSO_4_. (B) Pulsed-field gel electrophoresis analysis reveals that a wild type-like chromosome 8 (approximately 550 kb) replaced the rearranged chromosomes (in the size range of 900 kb and 650 kb) in the evolved cell populations. Evolved cultures from generations 100, 200, 300 and 400 were examined. A gene (*YHL002W*) located on the left arm of chromosome 8 was used as a probe for Southern blotting. M, yeast chromosomal DNA from a standard laboratory strain.

Large-scale chromosomal rearrangements can spread through a population if they are beneficial. However, the rearranged chromosomes can be quite unstable when growth conditions change and selective pressure is relieved [24], [49]. We set up a laboratory evolution experiment to investigate the stability of rearranged chromosomes. Five individual colonies derived from an EC9 haploid segregant carrying rearranged chromosomes (EC9-7 in Figure 3) were used to set up 10 independent evolving lines, 5 with relaxed selection (YPD containing 1 mM CuSO_4_) and 5 with strong selection (YPD containing 5 mM CuSO_4_). We regarded the medium containing 1 mM CuSO_4_ as a more relaxed growth condition because EC9 haploid segregants without rearranged chromosomes (such as EC9-6 in Figure 3B) could grow efficiently under such conditions. These cells were grown and diluted daily in fresh medium. After 400 generations, we observed that evolved cells with relaxed selection all exhibited improved growth on YPD or YPD with 1 mM CuSO_4_, but decreased tolerance to high concentrations of copper (Figure 6A).

We examined the karyotype of evolved cells collected from generations 100, 200, 300 and 400. In all five evolved cultures with relaxed selection, the rearranged chromosomes (in the size range of 900 kb and 650 kb) were replaced by a novel wild type-like chromosome 8 (approximately 550 kb) during the course of evolution (Figure 6B). Since each evolved culture was initiated from an independent single colony, it is likely that the novel chromosome 8 repeatedly evolved at least five times in our evolution experiment. In addition, this novel chromosome 8 could be detected in the populations collected from generation 100, suggesting that it already existed in the population at a very early stage of our experiment (Figure 6B). In contrast, four out of five evolved cultures under strong selection pressure retained the rearranged chromosomes in the majority of populations even after 400 generations (Figure S5A). We also set up evolution experiments using EC9 diploid cells. Similar results were observed except that it took a longer time to fix the wild type-like chromosome 8 in diploid populations (Figure S5B). One possible explanation for the difference between haploid and diploid populations is that the cost of carrying extra chromosomal fragments is relatively lower in diploid cells [50]. Together, these results suggest that these chromosomal rearrangements are highly dynamic and reversible.

To further investigate its structure, the novel 550-kb chromosome 8 of the evolved cells was purified from PFGE gels and subjected to array-based comparative genomic hybridization (aCGH). The result showed that the novel chromosome 8 had almost the same gene content as the wild type chromosome 8 except for some telomeric genes; *YHR217C*, *YHR218W* and *YHR219W* in the right telomere and *YHL044W*, *YHL045W*, *YHL048W*, *YHL049C* and *YHL050C* in the left telomere were undetected in our assay. On the other hand, we detected signals of other telomeric regions, including *YAR062W*, *YAR064W*, *YAR066W*, *YAR068W*, *YAR069C*, *YAR070C* and *YAR073W* from the telomere of chromosome 1 and *YFL065C*, *YFL066C* and *YFL067W* from the telomere of chromosome 6. Telomeric regions have been known to be very dynamic. It is possible that recombination between different telomeres occurred during our evolution experiment.

## Discussion

### Adaptive phenotypes can be generated by chromosomal rearrangements in natural populations

It has been observed that yeast can adapt to various nutrient-limited conditions [15], [44], [51]. Recent surveys on yeast strains collected from different continents also revealed that *S. cerevisiae* populations exhibit a high degree of phenotypic variance, suggesting that they have adapted to diverse ecological niches [26], [52]. However, unlike experimentally evolved cells, adaptations in natural populations are more difficult to study. It has remained elusive whether the types of mutations commonly observed in laboratory adaptation are also involved in natural adaptation.

Copper is an essential cofactor for many enzymes such as the cytochrome c oxidase in the respiratory chain. Nonetheless, an excess of copper is deleterious to cells [53]. The toxicity of copper may come from the generation of reactive oxygen species, the competition with other metals for their native binding sites, the alteration of protein conformations or interference with biochemical reactions [53], [54]. Cells have evolved multiple mechanisms to regulate copper homeostasis including different metal transporters, sequestration factors, and detoxification enzymes [54], [55].

The EC-C1 strains carry two rearranged chromosomes that significantly enrich the copy number of genes involved in copper regulation. Evolved phenotypes often arise from duplicated chromosomal fragments that contain the critical genes for adaptation in experimental yeast populations [14], [15], [44]. We speculate that the rearranged chromosomes in EC-C1 strains might result from selection for higher copper tolerance for the following reasons. First, among all the diploid yeast strains collected from Evolution Canyon, the EC-C1 strains constitute a major group (8/21 or 38%). In addition, the EC-C1 strains show very low levels of polymorphism in their microsatellite loci compared with other EC groups [43], suggesting that EC-C1 strains carry some adaptive phenotypes allowing them to quickly spread in Evolution Canyon. Second, when the copper content in the soil samples collected from different sites of Evolution Canyon was measured using inductively coupled plasma-atomic emission spectroscopy (see Materials and Methods), we found that the copper levels of most EC sites are above 30 ppm (with an average of 38 ppm) and in one area it even reaches 95 ppm, which are higher than the average copper content (20 ppm) in soil [56]. Previous studies have suggested that increased copper levels in vineyard soil caused the wine yeast strains to evolve higher copper tolerance [57], [58]. A similar adaptive process might also occur in the EC-C1 strains. Third, a previous study has shown that increased expression of *CUP1* also enhanced cadmium resistance of cells. When we examined cadmium sensitivity of twenty EC9 haploid segregants, the cadmium-resistant phenotype was not co-segregated with rearranged chromosomes. This suggests that increased copies of *CUP1* and *CUP2* were not a result of selection for the pleiotropic effect on cadmium resistance (Figure S6). In our lab, we have examined the fitness of all EC strains under more than 30 different growth conditions (including different temperatures, nutrient starvation, chemicals and metal ions). Only in two conditions, medium containing either high levels of copper or cadmium, EC-C1 strains showed higher growth rates ([38] and our unpublished results). Nonetheless, we cannot completely rule out the possibility that chromosomal rearrangements in EC-C1 strains were caused by adaptive effects resulting from other amplified genes or other unknown pleiotropy of Cup1 and Cup2. In the future, it will be interesting to compare the whole-genome gene expression patterns between haploid cells carrying rearranged or wild type chromosomes under different conditions. If differentially regulated genes are enriched in biological pathways other than copper tolerance, it may provide us a clue to further test other possible causes of chromosomal rearrangements in EC-C1 strains.

Increased *CUP1* copy number has been observed in copper-resistant strains isolated from laboratory evolution experiments, industry or natural habitats [59]–[61]. Nonetheless, EC-C1 diploid cells carry more than 20 copies of *CUP1* that are much higher than the *CUP1* amplification reported in previous cases (ranging from 2 to 15 copies). In addition, we showed that increased *CUP1* copy number alone was not enough to achieve the high copper tolerance observed in EC-C1 cells. Amplification of the copper-binding transcriptional factor *CUP2* was also critical, suggesting that a more complex adaptive strategy has occurred in EC-C1 strains. Increasing the dosage of a transcriptional factor may influence the expression of its downstream target genes to different levels depending on its feedback regulation or other compensatory mechanisms. Although the *CUP1* amplification clearly plays a major role in copper tolerance of EC-C1 cells, other downstream targets of Cup2 probably also contribute to the observed phenotype.

By combining the whole-genome gene expression analysis of cells carrying different copy numbers of *CUP2* and the functional assay, we identified and confirmed three previously unidentified genes, *PHO84*, *SCM4* and *CIN2*, that were involved in copper tolerance. In two of them (*SCM4* and *CIN2*) we also observed a conserved Cup2-binding motif sequence in their promoters. *CIN2* encodes a GTPase-activating protein involved in tubulin folding [62], and *cin2Δ* mutant cells are also sensitive to another metal, arsenic, suggesting that Cin2 is involved in metal regulation [63]. *SCM4* was previously identified as a suppressor of a cell cycle mutant of *CDC4* [64]. Nonetheless, the Scm4 protein contains four transmembrane domains and localizes to the mitochondria, an organelle involved in many metal metabolic pathways. It will be interesting to determine whether Scm4 affects copper tolerance through its function in mitochondria. *PHO84* encodes a high-affinity inorganic phosphate transporter that also functions in manganese homeostasis [65], [66]. The enhanced tolerance of *pho84Δ* mutants to several metal ions (including manganese, zinc, cobalt and copper) has been attributed to defects in the uptake of metal ions [66]. However, we found that deletion of *PHO84* in EC-C1 strains decreases tolerance to high concentrations of copper in a Cup1-dependent manner. This suggests that genetic background may have a strong influence on the regulatory network of metal metabolism.

### Dynamic large-scale chromosomal rearrangements in a fluctuating environment

Large-scale chromosomal rearrangements can quickly change the expression level of multiple genes or even a whole pathway by changing the gene copy number. In addition, the spontaneous rate of chromosomal rearrangements is higher than the spontaneous rate of point mutations [21]. This class of mutations is most likely to be found at the early stage of adaptation since they allow a brute force change in phenotype by changing multiple genes in one step. Many such examples have been reported in short-term experimental evolution in *S. cerevisiae* and *C. albicans* [8]–[11], [15], [24], [67]. But in addition to increasing the copy numbers of beneficial genes, rearrangement also increases copy numbers of other genes in the same chromosomal segments that may not be beneficial. It has been shown that when compared with euploid cells, aneuploid cells have higher fitness under certain conditions but have reduced fitness in general [50], [68]. Thus, chromosomal rearrangement is unlikely to be an optimal form of mutation, but may allow a population to survive until either better mutations appear or until a population's environment becomes more permissive. In the EC-C1 strains, we examined two loci on the rearranged chromosomes 7 and 8 (∼7 kb/each) and found that they had identical sequences as wild type chromosomes, supporting the idea that the rearranged chromosomes were recently generated rather than an ancient relic. A lingering question is then how cells adjust to the cost of these crude adaptive changes on longer evolutionary timescales, especially in a fluctuating environment.

When EC-C1 cells carrying the rearranged chromosomes were propagated in medium containing 1 mM copper sulfate, a wild type-like chromosome 8 quickly became fixed in all five individual populations in as early as 200 generations. The repetitive appearance of this novel chromosome 8 suggests that some large-scale chromosomal rearrangements are highly dynamic and reversible. This result is in agreement with a previous study showing that large-scale inter- and intra-chromosomal duplications were intrinsically unstable when no selective advantages were provided by those duplications [49]. Previous studies in budding yeast suggested that clustered Ty sequences might serve as double-strand break hotspots to initiate ectopic recombination in the yeast genome [28], [30]. This type of recombination allows cells to quickly adapt to stressful environments by duplicating chromosomal fragments that contain critical genes. Furthermore, when the stress is relieved or better mutations have evolved, the duplicated chromosomal fragments can revert back to the original configuration at a high frequency by another round of ectopic or homologous recombination. Such genome flexibility enables organisms to generate switch-like adaptive phenotypes. This would be especially valuable for sexual populations since large-scale chromosomal rearrangements often cause gamete lethality when they are heterozygous. This idea is indirectly supported by the observation that although large-scale chromosomal rearrangements occur frequently in laboratory evolution experiments or natural isolates, closely related yeast species such as *S. cerevisiae* and *S. paradoxus* still maintain colinear genomes [69]. Together with the facts that transposon expression is known to be activated under environmental stress and elevated transcription levels increase the rate of mitotic recombination [70]–[72], the abovementioned Ty-mediated chromosomal rearrangements supply the population with an effective mechanism to quickly respond to environmental changes.

## Materials and Methods

### Strains and genetic procedures

All EC diploid strains are *Saccharomyces cerevisiae* collected from an east-west facing canyon (Evolution Canyon) at Lower Nahal Oren, Israel [43]. In brief, EC33, 34, 35 and 36 were isolated from the south-facing slope (SFS), EC9, 10, 39 and 45 from the valley bottom (VB), and EC13, 57, 58, 59, 60 and 63 from the north-facing slope (NFS). Substitutive and integrative transformations were carried out by the lithium acetate procedure [73]. Media, microbial and genetic techniques were performed as described [74].

### Karyotyping of EC strains and Southern blot

A total of 1∼2×10^8^ yeast cells were used for plug preparation. Cells were washed with 1 ml EDTA/Tris (50 mM EDTA, 10 mM Tris, pH 7.5) and transferred into EDTA/Tris with 0.13 mg/ml zymolyase (Seikagaku America Inc., St. Petersburg, FL). The cell mixtures were incubated for 30 s at 42°C and then embedded in low melting point agarose (Sigma-Aldrich, St. Louis, MO). The agarose plugs were placed at 37°C overnight for zymolyase digestion. After digestion, the agarose plugs were placed in LET solution (0.5 M EDTA, 10 mM Tris, pH 7.5) containing 2 mg/ml protease K and 1% N-lauroylsarcosine at 50°C overnight. This step was repeated three times. The plugs were transferred to EDTA/Tris solution and dialyzed four times for 1 h at 37°C. Yeast chromosomes were separated on 0.7% agarose gels by pulsed field gel electrophoresis (PFGE) using a Rotaphor Type V apparatus (Biometra, Göttingen, Germany). Electrophoresis was performed for 48 h at 13°C in 0.5× TBE buffer at a fixed voltage of 120 V and an angle of 115° with pulse time intervals of 30 s.

After PFGE, the chromosomal DNA was depurinated and denatured by incubating the agarose gel in 0.25 N HCl and then in alkaline solution (0.5 M NaOH, 1.5% NaCl). The DNA was transferred to a charged nylon membrane, Immobilon-NY+ (Millipore, Billerica, MA). DNA probes for each chromosome were obtained by PCR using the primers listed in Table S1. The Digoxigenin-labeled DNA probes were prepared using the DNA labeling and detection kit (Roche, Indianapolis, IN).

### Array-based comparative genomic hybridization

Oligonucleotide arrays were produced at the Microarray Core, Institute of Molecular Biology, Academia Sinica, using an Omnigrid 100 arrayer (Digilab, Holliston, MA) and the Yeast Genome Array-Ready Oligo Set (Version 1.1, Operon, Huntsville, AL). The printing protocol can be found at the Institute of Molecular Biology Microarray Facility web site (http://www.imb.sinica.edu.tw/mdarray/methods.html).

Yeast genomic DNA was extracted using the Qiagen Genomic-Tip 100/G kit (Qiagen, Valencia, CA). For individual-chromosome aCGH, DNA was excised from PFGE gel after EtBr staining and purified using the Geneaid Gel DNA Fragment Extraction kit (Geneaid, Taiwan). The purified chromosomal DNA was further amplified using GenomePlex Whole Genome Amplification Kit (Sigma-Aldrich, St. Louis, MO). Probe preparation and hybridization were performed as described [75]. The array data were analyzed using GeneSpring GX 7.3.1 (Agilent, Santa Clara, CA).

### Quantitative PCR

After copper treatment (1 mM CuSO_4_) for 2 h at 28°C, total RNA was isolated by Qiagen RNeasy Midi Kit (Qiagen). First-strand cDNA was synthesized for 2 h at 37°C using the High Capacity cDNA Reverse Transcriptase Kit (Applied Biosystems, Foster City, CA). A 20-fold dilution of the reaction products was then subjected to real-time quantitative PCR using gene-specific primers, SYBR Green PCR master mix and an ABI-7000 sequence detection system (Applied Biosystems). Data were analyzed using the built-in analysis program.

### Fitness assays

Fitness of individual strains was obtained by propagating replicate cultures in complete synthetic medium (CSM) with or without 1.5 mM CuSO_4_ in 96-well plates inside a temperature controlled, shaking plate reader Infinite F200 (Tecan, Mannedorf, Switzerland). Growth rates were calculated as the maximum slope that could be derived from any continuous 2-hour period during the 20-hr assay. Four replicate cultures were used per strain.

To determine competitive relative fitness, we measured the fitness of the experimental strains by competing them against a reference strain expressing PGK1::GFP in YPD media at 28°C. The testing cells and reference cells were inoculated in the YPD medium individually and acclimated for 24 h. The cells were subsequently diluted in fresh media and incubated for another 4 h. The reference and testing cells were then mixed (1∶1 ratio), diluted into fresh medium at a final cell concentration of 5×10^3^ cells/ml, and allowed to compete for 17 h, which represents about 11 generations of growth. The ratio of the two competitors was quantified at the initial and final time points using a fluorescence activated cell sorter (FACSCalibur, Becton Dickinson, Franklin Lakes, NJ). Four independent replicates for each fitness measurement were performed.

### Rearranged chromosome sequencing

We sequenced both wild type and rearranged chromosomes. To prevent cross-contamination between wild type and rearranged chromosomes, wild type chromosomes were purified from EC9-8 haploid cells that do not carry any rearranged chromosome, and rearranged chromosomes were purified from EC9-7 haploid cells that do not carry wild type chromosome 8 (Figure 3). Individual chromosomal DNA was purified from PFGE gels. Two regions on chromosome 7 (from 188,179 to 194,679 bp) and chromosome 8 (from 496,154 to 502,755 bp) were amplified by PCR using a set of primers. The PCR products were purified and then sequenced. The accession numbers for the sequences are JN835223 and JN835224.

To identify the junction sites of the rearranged chromosomes, we designed primers near each possible breakpoint (*YHR015W*, *YHR210C*, *YHL008C*, *YHR219W*, *YHL050C*, *YHR145C*, *YGL200C* and *YGL096W*) according to the aCGH data. For each rearranged chromosome, four different combinations of primer pairs were used to PCR the junction site. Only one pair of the primers could successfully amplify the junction site. The PCR products were purified and then sequenced. The accession numbers for the sequence are JX101633 and JX101634.

### Whole-genome expression analysis

For the whole-genome gene expression analysis, log-phase cells were grown in YPD with 1 mM CuSO_4_ (which does not affect the growth of *cup2* null mutant cells) for 1 h. Total RNA from the treated samples and the corresponding untreated control samples (in YPD) was isolated using the Qiagen RNeasy Midi Kit (Qiagen). Probe preparation and hybridization were performed as described [75]. The array data were analyzed using GeneSpring GX 7.3.1 (Agilent). We excluded the data with hybridization intensities lower than 500 as they were close to the background values (∼200). The intensities of each array were normalized using a LOWESS function [76].

### Experimental evolution

EC9-7 haploid cells were streaked out on a YPD plate to form single colonies. Five individual colonies (A1–A5) were then used to initiate the evolution experiment. Cells were cultured in 3 ml YPD with 1 mM CuSO_4_ (E1–E5) or YPD with 5 mM CuSO_4_ (E6–E10) through a daily 1000-fold dilution (about 10 generations). Once every five transfers population samples from each line were stored in 20% glycerol at −80°C for later analysis. For diploid evolution experiments, EC9 diploid cells were diluted and plated on an YPD plate to grow to single colonies. Five individual colonies (AD1–AD5) were then used to initiate the evolution experiment (ED1–ED5) with a protocol similar to that of haploid evolution experiments.

### Measurement of soil copper concentrations

Soil samples were collected at 7 locations of Evolution Canyon corresponding to the collection sites of the EC yeast strains (three at the SFS, one at the VB, and three at the NFS). Copper contents in soil were measured by inductively coupled plasma-atomic emission spectroscopy (ICP-AES) using at least 200 g of individual samples.

### Data access

The array CGH data are available from the NCBI Gene Expression Omnibus (GEO) (http://www.ncbi.nlm.nih.gov/geo/) under accession numbers GSE22431, GSE38034 and GSE33652. The expression data are available from GEO under accession number GSE31661.

## Supporting Information

Figure S1 EC-C1 strains contain three rearranged chromosomes. (A) Three different chromosomes were hybridized in EC-C1 strains when a gene (*YHR003W*) located on the right arm of chromosome 8 was used as a probe for Southern blotting. A similar pattern was observed when two other genes (*YHL002W* and *YHR102W*) were used as probes. (B)(C) Two different chromosomes were hybridized in EC-C1 strains when a gene (*YHL027W*) located on the left arm of chromosome 8 or a gene (*YGL175W*) located on the left arm of chromosome 7 were used as a probe for Southern blotting. The 650-kb chromosome was hybridized by both probes. (D) EC-C1 strains contain an enlarged chromosome 10. A gene (*YJL001W*) located on the left arm of chromosome 10 was used as a probe for Southern blotting. A similar pattern was observed when another gene (*YJR001W*) was used as a probe.
(EPS)

Figure S2 Aligned DNA sequences of the junction sites. (A) The junction site sequence of the rearranged 900-kb chromosome. (B) The junction site sequence of the rearranged 650-kb chromosome. Ty1, a Ty1 sequence found only in the junction site. DNA sequences of the flanking regions can be found in GenBank under the accession numbers JX101633 and JX101634.
(EPS)

Figure S3 Haploid segregants of EC9 carrying rearranged chromosomes are more tolerant to copper. (A) Segregants carrying the rearranged chromosomes are more copper-tolerant. Haploid segregants from four-viable-spore tetrads of EC9 strains were grown in YPD media overnight, serially diluted and plated on YPD plates containing different concentrations of CuSO_4_. Plates were incubated at 28°C until obvious colonies were formed. (B) Pulsed-field gel electrophoresis analysis of haploid segregants from four four-viable-spore EC9 tetrads. In four haploid segregants from a single tetrad, two of them carry two rearranged copies of chromosome 8, and the other two carry a wild type copy of chromosome 8. A gene (*YHL002W*) located on the left arm of chromosome 8 was used as a probe for Southern blotting. M, yeast chromosomal DNA from a standard laboratory strain.
(EPS)

Figure S4 *pho84*, *scm4*, and *cin2* mutant cells are sensitive to copper but not sulfates. Results in Figure 5 showed that *pho84*, *scm4* and *cin2* mutant cells are sensitive to CuSO_4_. To rule out the possibility that these mutants are sensitive to sulfates instead of copper, the same set of mutant cells were tested on YPD plates containing different concentrations of CuCl_2_.
(EPS)

Figure S5 Rearranged chromosomes are more likely to revert back to wild type-like chromosomes only in cultures with relaxed selection. (A) Most of the haploid cells evolving under strong selection (E6–E10) maintain the rearranged chromosomes even after 400 generations. (B) In diploid populations evolving under relaxed selection (ED1–ED5), a wild type-like chromosome 8 (approximately 550 kb) replaced the rearranged chromosomes (in the size range of 900 kb and 650 kb). Evolved cultures from generations 100, 200, 300 and 400 were examined. A gene (*YHL002W*) located on the left arm of chromosome 8 was used as a probe for Southern blotting. M, yeast chromosomal DNA from a standard laboratory strain.
(EPS)

Figure S6 Rearranged chromosomes do not correlate with the cadmium-resistant phenotype. Twenty EC9 haploid segregants were grown in YPD media overnight, serially diluted and plated on YPD plates containing different concentrations of CdCl_2_. Plates were incubated at 28°C until obvious colonies were formed.
(EPS)

Table S1 Single nucleotide polymorphisms in the junction sites of EC-C1 strains.
(PDF)

Table S2 Gene expression in cells under copper treatment.
(PDF)
