# Supplementary material for: Dynamic Large-Scale Chromosomal Rearrangements Fuel Rapid Adaptation in Yeast Populations
Source: PLoS Genet. 2013 Jan 24;9(1):e1003232. doi: 10.1371/journal.pgen.1003232 (PMC3554576; doi:10.1371/journal.pgen.1003232)
Supplement: Table S1 — Single nucleotide polymorphisms in the junction sites of EC-C1 strains. (PDF) [file pgen.1003232.s007.pdf]

TABLE S1. Single nucleotide polymorphisms in the junction sites

Strain	Chr VII + VIII[a]		Chr VIII + VIII[a]	
EC10	1/455	0.22%	1/1166	0.09%
EC35	1/455	0.22%	1/1166	0.09%
EC36	0/455	0%	1/1166	0.09%
EC39	0/455	0%	1/1166	0.09%
EC57	0/455	0%	1/1166	0.09%
EC58	0/455	0%	1/1166	0.09%

[a] number of single nucleotide polymorphisms/number of total sequenced nucleotides in the junction site.
