# Supplementary material for: Dynamic Large-Scale Chromosomal Rearrangements Fuel Rapid Adaptation in Yeast Populations
Source: PLoS Genet. 2013 Jan 24;9(1):e1003232. doi: 10.1371/journal.pgen.1003232 (PMC3554576; doi:10.1371/journal.pgen.1003232)
Supplement: Table S2 — Gene expression in cells under copper treatment. (PDF) [file pgen.1003232.s008.pdf]

TABLE S2. Gene expression under copper treatment

Genes whose expression is up-regulated more than 1.5-fold (*CUP2, CUP2*/*cup2*Δ, *cup2*Δ)
PDR5, PHO84, GLN1, GLT1, SNQ2, ILV3, ACO2, CYB5, SCM4, ARG4, MET6, CLN2, YGL117W, ERG11, LYS4, GFD2, DUR1,2, CPA2, STP4, HPT1, HIS1, ARO10, MET10, ERG24, ASN1, RLI1, FCY2, YMC2, CIN2, YHR022C, LIA1, RFU1, SAM2, FUR1, ERG28, HNM1, SDH2, YPS3, FCY21
Genes whose expression is down-regulated more than 1.5-fold *(CUP2, CUP2/cup2*Δ, *cup2*Δ*)*
RPN3, GSH1, SCL1, PUT3, CDC48, PUP3, YFR035C, YBL107C, YME1, CUR1, MGA1, RPN11, ECM21, RRF1, RPN1, PDI1, PRE2, RAD52, PRE4, IRC20, MND1, FMP40, RPN9, YML131W, PRE3, RPT6, UME6, RPN6, ALG13, SLG1, PRE10, PRE8, PRE1, RPN7, DSK2, RPN2, YHR033W, CAD1, PRB1, YCR062W, NPL4, RPT1
